# Supplementary material for: Identification of microRNAs in the Lyme Disease Vector Ixodes scapularis
Source: Int J Mol Sci. 2022 May 16;23(10):5565. doi: 10.3390/ijms23105565 (PMC9141961; doi:10.3390/ijms23105565)
Supplement: Supplementary file 1 [file ijms-23-05565-s001.zip › ijms-1705369-supplementary.pdf]

International Journal of
Molecular Sciences

MDPI

Supplementary materials

Identification of microRNAs in the Lyme Disease Vector *Ixodes scapularis*

Deepak Kumar [1,2], Latoyia P. Downs [2], Monica Embers [3], Alex Sutton Flynt [1,2] and Shahid Karim [1,2,*]

1 Center for Molecular and Cellular Biosciences, University of Southern Mississippi, Hattiesburg, MS 39406, USA; deepak.kumar@usm.edu (D.K.); alex.flynt@usm.edu (A.F.)
2 School of Biological, Environmental, and Earth Sciences, University of Southern Mississippi, Hattiesburg, MS 39406, USA; latoyia.downs@usm.edu
3 Division of Immunology, Tulane National Primate Research Center, Covington, LA 70433, USA; members@tulane.edu

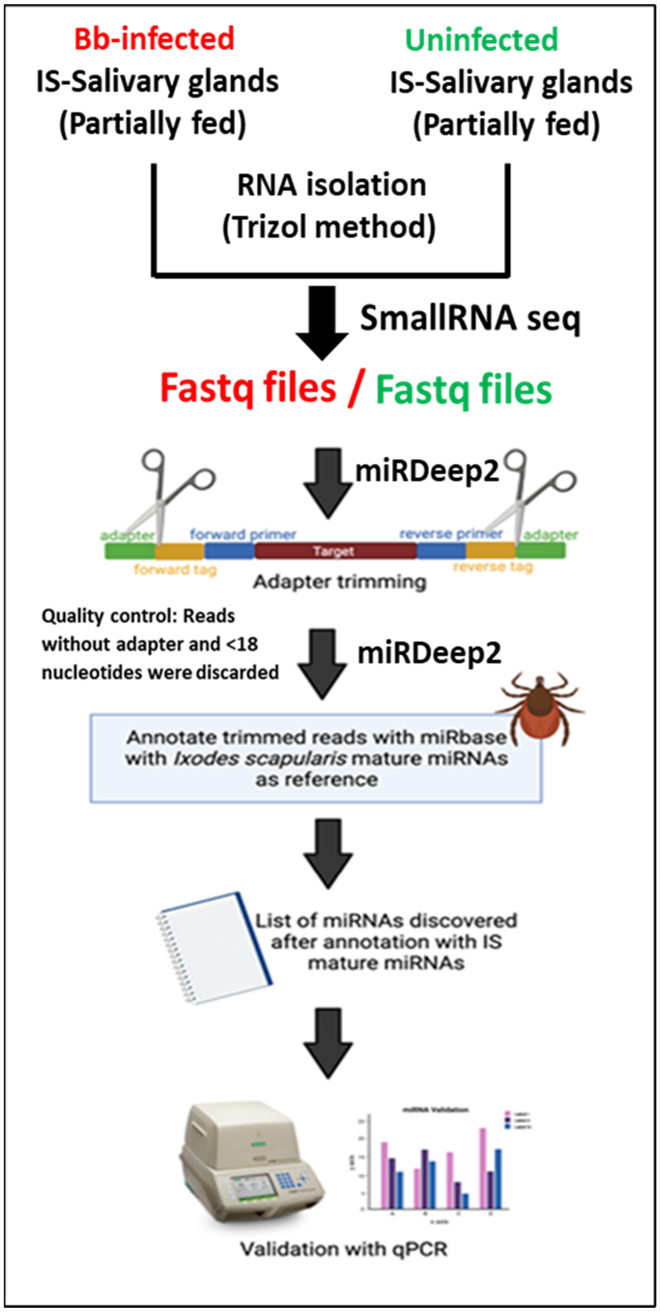

Figure S1. Schematic of the experimental plan and data analysis.

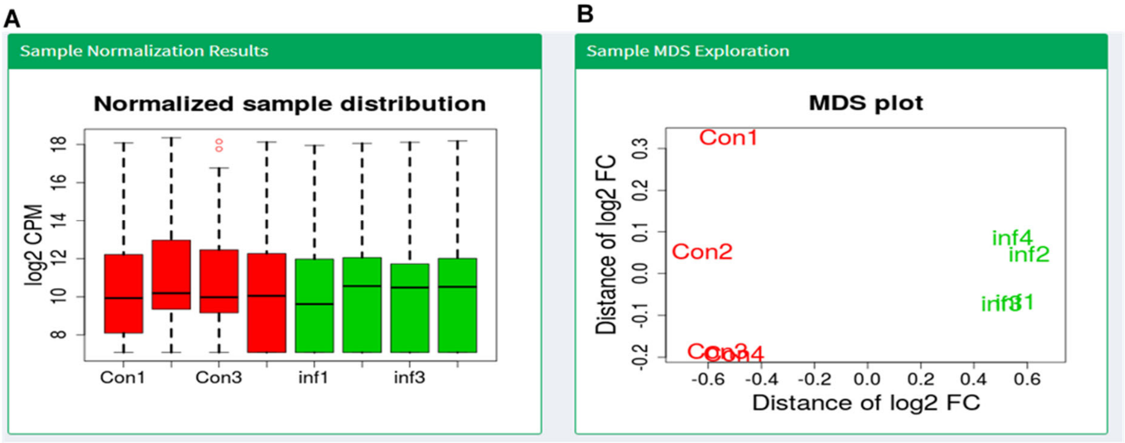

Figure S2. A. Normalized sample distribution for *B. burgdorferi*-infected (inf1, inf2, inf3, and inf4) and uninfected (Con1, Con2, Con3, and Con4) salivary glands. **B.** Multidimensional ccaling (MDS) plot shows variation in differential expression among uninfected samples (Con1, Con2, Con3, and Con4), where the distance between sample labels indicates dissimilarity in log2-fold change (FC).

Table S1. A. MicroRNA profiling of *Ixodes scapularis* salivary glands (novel miRNAs predicted by miRDeep2).

Provisional id	miRDeep2 Score	mature read count	miRbase miRNA with same seed	consensus mature sequence
isc-miR-4968-5p	1.80E+04	36306	dme-miR-4968-5p	aagcugcccaaugaagggcug
NW_002532706.1_43464 isc- miR-92a-3p	1.50E+04	29572	dme-miR-92a-3p	uauugcacucgucccggccuuu
NW_002871802.1_367 isc -miR-125-5p	1.20E+04	12052	dme-miR-125-5p	ucccugagacccuaacuuguga
NW_002692816.1_23152 isc-miR-6-3p	7.60E+03	10940	dme-miR-6-3p	uaucacagccaucuuugaugacc
nDs_002867181.1_816	2.40E+03	4514		gccaacccacuaacugacggga
nDs_002653147.1_27921	2.30E+03	4514		gccaacccacuaacugacggga
nDs_002794895.1_10282	2.30E+03	4514		gccaacccacuaacugacggga
nDs_002645436.1_28906	1.80E+03	3392		gcuguuaguuuguggggguggug
nDs_002860940.1_1699	1.80E+03	3392		gcuguuaguuuguggggguggug
nDs_002508325.1_46074	1.80E+03	3279		gcuguuaguuuguggggguggug
nDs_002699119.1_22404	1.70E+03	3392		gcuguuaguuuguggggguggug
NW_002767753.1_13364 isc-miR-958-3p	1.70E+03	3446	dme-miR-958-3p	ugagauucaacuccuccaacuucu
nDs_002667887.1_26137	1.70E+03	3392		gcuguuaguuuguggggguggug
nDs_002810888.1_8213	1.70E+03	3392		gcuguuaguuuguggggguggug
nDs_002642603.1_29251	1.70E+03	3392		gcuguuaguuuguggggguggug
nDs_002815928.1_7504	1.00E+03	2109		caagaagauagaaucaaugaga
nDs_002861213.1_1662	9.50E+02	1855		cuugggcuggaguucgucguugu
isc-miR-190-5p	6.60E+02	621	dme-miR-190-5p	agauauguuugauauucuugguu
nDs_002614457.1_32408	6.30E+02	1205		cacgucgaggcuucagguugu
nDs_002614457.1_32404	5.20E+02	844		uacgucgagaccucagguuga
nDs_002867165.1_832	5.00E+02	994		ucguuuucgagaucgugaaccu
nDs_002728031.1_18707	4.90E+02	952		aguggucaugucuucgcacugga
nDs_002621331.1_31664	4.50E+02	812		gugugagaaaaugguuggcaca
nDs_002614457.1_32406	4.50E+02	857		cacgucgagauuucaagcuaug

nDs_002735932.1_17756	4.10E+02	752		ugaggucaugcucggugccuug
nDs_002873519.1_126	3.70E+02	733		auaucuuuuuuuaacaucggcu
nDs_002784743.1_11607	2.10E+02	361		cauucuugcagcgaaggguugu
nDs_002620414.1_31747	1.50E+02	213		cagugcuucugcagugcaggc
nDs_002871485.1_401	1.40E+02	280		acagaaucgagcgcuagggcga
nDs_002668420.1_26105	1.10E+02	200		ggcacaaggcugucggaugcga
nDs_002680650.1_24606	1.00E+02	146		cucagaaaugcuaggcuaucgg
NW_002527183.1_43988 isc-miR-263b-5p	1.00E+02	184	dme-miR-263b-5p	cuuggcacugaaagaauucaca
nDs_002685244.1_24008	9.90E+01	185		cuuguuugggcaaaugggugac
nDs_002814339.1_7692	9.60E+01	185		cacgggccccagcugaacgcu
nDs_002737590.1_17488	9.00E+01	170		cagucaguggcuuugguucauc
nDs_002737673.1_17464	8.60E+01	173		guacacguucgggcuuccaccc
nDs_002564688.1_39220	7.40E+01	96		gguuauuuucagcggcugucgu
nDs_002871133.1_436	7.00E+01	146		agccauuuuuugaagucgaca
nDs_002737711.1_17449	4.80E+01	84		agagugacgcagagaacaaauc
nDs_002675644.1_25181	4.50E+01	84		aaaaggacagaacauguagaca
nDs_002763926.1_13886	3.90E+01	69		uguagccggauuguggacugg
nDs_002531612.1_43580	3.70E+01	53		cacugugcgcgauugguucaac
nDs_002722741.1_19330	3.30E+01	75		auugaacgagaggacauaguc
nDs_002567548.1_38945	3.10E+01	52		ucaguguccgucgccggaagg
NW_002787101.1_11264 isc-miR-31b-5p	3.10E+01	26	dme-miR-31b-5p	aggcaagaagucuuuuaggaug
nDs_002754008.1_15271	3.00E+01	70		ucacuuuacgucguaguacaag
nDs_002538194.1_42559	3.00E+01	53		uuaguacgauccgucgaaagac
nDs_002801682.1_9417	3.00E+01	54		uguagcaugcuccaagcggagg
nDs_002508917.1_46002	3.00E+01	54		uguagcaugcuccaagcggagg
nDs_002568676.1_38752	2.70E+01	48		aaggagacaaggaaacaaacacc
nDs_002642725.1_29244	2.60E+01	58		acaacccggaaucgucauggcc
nDs_002670695.1_25779	2.60E+01	40		gcuggaucguaaaacuggugcca
nDs_002721909.1_19420	2.40E+01	44		gagaggucgguagaguugcgc
nDs_002548557.1_41255	2.40E+01	40		cuagugugacugucacagugg
nDs_002856865.1_2668	2.40E+01	40		cccggcgagaaaucuggcggccg
nDs_002588255.1_36295	2.10E+01	24		agacacgcuacugacuaucgucg
nDs_002591771.1_35729	1.80E+01	12		cccgguacggacugcgcauguc
NW_002637235.1_29734 isc-miR-4987-5p	1.80E+01	20	dme-miR-4987-5p	augcaacagaggcgagaugagacg
nDs_002730915.1_18372	1.80E+01	21		gaggaagaaaacgcgaaaggac
nDs_002527216.1_43980	1.70E+01	26		aggugaaugaauacccagucgaug
nDs_002829027.1_5932	1.60E+01	15		acgaaaucccgcggaucgcauug
nDs_002741178.1_17075	1.60E+01	27		uugggcuuuaaacaucaugagc
nDs_002718594.1_19992	1.50E+01	25		ucacaggacgugcaacggugcu
nDs_002718594.1_19994	1.50E+01	25		ucacaggacgugcaacggugcu
NW_002681830.1_24464 isc-bantam-3p	1.40E+01	17	dme-bantam-3p	cgagaucauacgucgguggugg
nDs_002515969.1_45216	1.40E+01	17		acaauugaaguggaucccgaau
nDs_002770056.1_13170	1.40E+01	24		aacgaaucgaaggucaaagugc
nDs_002635366.1_29915	1.30E+01	36		cuugugaugcucucguucgacc
nDs_002745696.1_16495	1.20E+01	16		uccgaggaaguguccugacaga
nDs_002633080.1_30207	1.10E+01	17		uauuccuuccuuuuguccgguc
nDs_002862093.1_1546	1.00E+01	18		cuauuggacgaaaaggauagcu
nDs_002770523.1_13144	8.5	11		ugaagaucuccaacuuggccucg
nDs_002637837.1_29679	8	10		aaagaaauacggacgacaggag
NW_002716687.1_20192 isc-miR-5-5p	5.7	209	dme-miR-5-5p	caaggaaccgacgaaucgagugu

NW_002522525.1_44526 isc-miR-1007-5p	5.6	30	dme-miR-1007-5p	ucaguguuuggcuggaacccg
NW_002784743.1_11604 isc-miR-277-3p	5.4	1487	dme-miR-277-3p	uaaaugcauuaucuggaaug
NW_002788893.1_11074 isc-miR-4957-3p	5.3	52	dme-miR-4957-3p	ucagcugcgugaacgcgggcgcc
NW_002799140.1_9764 isc-miR-2a-1-5p	4.5	101	dme-miR-2a-1-5p	uucucaaauguccgccacucg
nDs_002745459.1_16501	2.7	25		accccgggggguggucacgugac
nDs_002813294.1_7883	2.5	19		acggagcgacugaacaagaca
nDs_002829114.1_5911	2.4	19		agagaaaaagacagguagaacg
nDs_002759900.1_14325	2.4	95		uugccgccgucguucauggaugg
nDs_002815075.1_7597	2.4	98		uugccgccgucguucauggaugg
nDs_002513235.1_45498	2.3	26		ccucgguuuucggcuggcacucg
nDs_002676299.1_25117	2.3	22		ccaccgguuacgcucugcgcc
nDs_002700913.1_22252	2.3	123		gggacgaaacagacgacacagc
nDs_002684507.1_24073	2.3	258		caggcgggagaacaauguccu
nDs_002756339.1_14956	2.3	13		caagucgcggcugcacgggcgcc
nDs_002774760.1_12785	2.3	12		cuucucucugcugccguggccc
nDs_002696292.1_22736	2.3	39		cugccucgcgcuggucuccgcu
nDs_002550131.1_40913	2.2	19		cgccucagcuguuucugcucc
nDs_002542368.1_42013	2.2	18		cacgucacagcguccgcucaag
nDs_002586787.1_36439	2.2	37		uuggacgaaaaaaagagccgacu
nDs_002607638.1_33547	2.2	17		cuagccaaucguggcggucggc
nDs_002836532.1_4888	2.2	22		ccaccgguuacgcucugcgcc
nDs_002597262.1_35126	2.1	11		cgggaucucggaggccaugaa
nDs_002662116.1_26803	2.1	21		caggauccugacgcuggugggcaca
nDs_002636820.1_29767	2.1	7545		caaggcaaugaacaugaucuca
nDs_002799981.1_9646	2.1	24		uccguccguuccgucccucu
nDs_002612646.1_32606	2.1	7545		caaggcaaugaacaugaucuca
nDs_002629651.1_30593	2.1	819		cuucguagucggauuaugacu
nDs_002601819.1_34393	2.1	32		ucgaccgcucgccacggcugg
nDs_002540798.1_42197	2.1	20		caugucacugcguccgcgcaag
nDs_002654127.1_27778	2	200		uuuccgcugucgcuuguagaug
nDs_002860687.1_1738	2	15		gcagaucucagguguagugauc
nDs_002665175.1_26443	2	217		aacuucgugcugcaggagcgccu
nDs_002751908.1_15689	2	49		uaauuggucuuugugagugcuu
nDs_002543222.1_41886	2	149		gccggugucgaucuugaaguuc
nDs_002569324.1_38643	2	22		ccaccgguuacgcucugcgcc
nDs_002590536.1_35849	2	22		uucgguugugccgcuugccu
nDs_002601819.1_34394	2	32		ucgaccgcucgccacggcugg
nDs_002792777.1_10524	2	15		ugagaucucggaggucgugcgcc
nDs_002714141.1_20473	2	83		ggcuucguagucggauuauga
nDs_002744939.1_16574	1.9	829		cuucgaaaucggauuaugacu
nDs_002533105.1_43443	1.9	7545		caaggcaaugaacaugaucuca
nDs_002753934.1_15280	1.9	1842		cauccgguccuaagaagucgaa
nDs_002692659.1_23173	1.9	11		cugccgaaguagcgucugcucu
nDs_002733141.1_18085	1.9	7545		caaggcaaugaacaugaucuca
nDs_002816672.1_7394	1.9	200		uuuccgcugucgcuuguagaug
nDs_002738551.1_17311	1.9	77		cgcgcggacgcugugacgcagc
nDs_002523780.1_44319	1.9	7545		caaggcaaugaacaugaucuca
nDs_002828015.1_6065	1.9	2093		ccaggauuugaacucugggccu
nDs_002829464.1_5811	1.9	7545		caaggcaaugaacaugaucuca
nDs_002545827.1_41513	1.9	44		aagggaaccgugcgagagcugcu

nDs_002692659.1_23171	1.8	11		cugccgaaguagcgucugcucu
nDs_002661855.1_26902	1.8	70		gacauaguaaggauugacg
nDs_002765967.1_13675	1.8	17		uauuccuuccuuuuguccgguc
nDs_002574390.1_37989	1.8	259		uuccgccgucgaucguagaug
nDs_002550131.1_40914	1.8	19		cgccucagcuguuucugcucc
nDs_002871802.1_364	1.8	103		agggccugagaaucuaaccugg
nDs_002673151.1_25529	1.8	29		gcgcacagagagacgaagacug
nDs_002809300.1_8415	1.8	1873		cauccgguccuaagaagucgaa
nDs_002738154.1_17397	1.8	35		cggaaggauaguggguggaccug
nDs_002532161.1_43535	1.8	1943		cauccgguccuaagaagucgaa
nDs_002798374.1_9856	1.8	271		uucgagcgcuaggacagaggccg
nDs_002724962.1_19022	1.8	339		agaaagugcgucuggcggcg
nDs_002692659.1_23176	1.8	108		acuaaaaaacaacggacaagu
nDs_002744911.1_16578	1.8	11		auucggacauccgcaggacguc
nDs_002545470.1_41626	1.8	11		auucggacauccgcaggacguc
nDs_002785931.1_11413	1.8	7545		caaggcaaugaacaugaucuca
nDs_002685244.1_24007	1.8	185		cuuguuugggcaaaugggugac
nDs_002784743.1_11583	1.7	12		aggacgauugagcucgac
nDs_002587969.1_36333	1.7	53		cacgauugccaaacuguaugcu
nDs_002661304.1_26957	1.7	39		aguccgucgucgaucuagccu
nDs_002707270.1_21452	1.7	26		uucgcguucggacguguucgaa
nDs_002549652.1_41058	1.7	1892		caugccguggcuugacca
nDs_002737099.1_17587	1.7	379		uuuggcaggcuuagaaucacuc
nDs_002511156.1_45736	1.7	95		uuugcaccgucgcucguagaug
nDs_002608667.1_33381	1.7	30		auggacuugauguuuuaggcucagu
nDs_002699570.1_22364	1.7	108		acuaaaaaacaacggacaagu
nDs_002537755.1_42809	1.6	53		acaccgguauuuaagagguuaucug
nDs_002553320.1_40621	1.6	11		auucggacauccgcaggacguc
nDs_002581904.1_37014	1.6	12		gccauagcguaacagagugacg
nDs_002541749.1_42092	1.6	1943		cauccgguccuaagaagucgaa
nDs_002509761.1_45913	1.6	74		gacuucagguccaucuggacg
nDs_002542368.1_42015	1.6	18		cacgucacagcguccgcucaag
nDs_002650258.1_28240	1.6	56		gcuguuaguuugugggauggu
nDs_002745745.1_16473	1.6	259		uuccgccgucgaucguagaug
nDs_002692816.1_23143	1.6	105		uuucacagucccuuugacgg
nDs_002664372.1_26487	1.6	18		acuaccgaccgugugugaccg
nDs_002677543.1_24907	1.6	1862		cauccgguccuaagaagucgaa
nDs_002505804.1_46391	1.6	74		gacuucagguccaucuggacg
nDs_002855841.1_2713	1.6	137		acugugauugaacugaacgaca
nDs_002592079.1_35704	1.6	14		gaguagaaugccugaauguugcu
nDs_002653831.1_27809	1.6	114		aaggcgaacgcugaccugggcc
nDs_002630829.1_30421	1.5	17		cgacgaauuucuggagaucgca
nDs_002535729.1_43080	1.5	331		cuucgcucugcuaaaguggacc
nDs_002590762.1_35829	1.5	95		uugccgccgucguucauggaugg
nDs_002617786.1_32035	1.5	18		ucugucccugguuuuggcgcu
nDs_002834843.1_5113	1.5	21		cgagggcuguccagaguucuug
nDs_002844434.1_4087	1.5	258		uuccgccgucgaucguagaug
nDs_002838169.1_4671	1.5	47		accauuugggucucuguccucu
nDs_002567550.1_38944	1.5	97		uuugcaccgucgcucguagaug
nDs_002639690.1_29492	1.5	2928		guuguacccagucgucgauguc
nDs_002530398.1_43703	1.4	31		cagggccuaaaugacauuuacacu
nDs_002829616.1_5794	1.4	15		acggcuggcgucucgcugcacc
NW_002626049.1_30948 isc-miR-981-3p	1.4	4936	dme-miR-981-3p	uucguugucguagaaaccugau

nDs_002790353.1_10903	1.4	20		cacgucacagcguccgcucaag
nDs_002721909.1_19415	1.4	312		caugacugucaucuuugcaucu
nDs_002592028.1_35707	1.4	26		cucgaggucuacccgcucuccu
nDs_002697784.1_22512	1.4	121		uuggacuuugagcauggcgaagg
nDs_002515625.1_45241	1.3	24		augcccacugucccaguuucugc
nDs_002815935.1_7502	1.3	15		ucugcgagacgcucaacgacag
nDs_002650258.1_28243	1.2	23		cucaagaucuucggccagucgc
nDs_002663292.1_26641	1.2	137		acugugauugaacugaacgaca
nDs_002744911.1_16577	1.2	11		auucggacauccgcaggacguc
nDs_002827450.1_6124	1.2	20		cacgucacagcguccgcucaag
nDs_002545470.1_41625	1.2	11		auucggacauccgcaggacguc
nDs_002553320.1_40622	1.2	11		auucggacauccgcaggacguc
nDs_002598280.1_35014	1.1	28		ucaacuccuucagcuggugcug
nDs_002756375.1_14945	1.1	52		uucacgaaucugaacgggaca
nDs_002613137.1_32572	1.1	19		ugugaaggccuccugugcucu
nDs_002563762.1_39378	1	18		aagaauaaacaaagguacggau
nDs_002768943.1_13309	0.9	121		ugacgucaucguagugcugcgu
nDs_002546088.1_41480	0.8	18		aucuuuugacacguuggaucu
nDs_002856883.1_2662	0.8	38		ugugagaugauuguugcauaagcg
nDs_002615653.1_32278	0.8	257		uuccgccgucgaucguagaug
nDs_002655152.1_27700	0.8	321		ugaaagcgaguaccaugacugu
nDs_002808312.1_8698	0.6	59		uuguggacgccuggacaagccu
nDs_002784518.1_11647	0.5	26		aucuuuugacacguuggaucu
nDs_002860612.1_1764	0.4	11		aacaugagcgacgucgcuaccg
NW_002847019.1_3755 isc-miR-1000-3p	0.3	49	dme-miR-1000-3p	augcuggggacacugaaaucc
nDs_002585048.1_36632	0.3	9		cuucguagucggauuauga
nDs_002569020.1_38698	0.2	353		gccggaacagucaucguugcug
nDs_002820844.1_6987	0.2	39		auucggcggcacuuugaacgaa
NW_002573167.1_38165 isc-miR-2494-3p	0	79	dme-miR-2494-3p	uucccaguagucccaggaccug

Table S1. B. mature miRNAs of *Ixodes scapularis* (already available in miRBase) detected in miRDeep2 analysis.

tag id	miRDeep2 score	mature read count	mature miRBase miRNA	consensus mature sequence
NW_002860020.1_1824	6.60E+05	1296028	isc-miR-375	uuuguucguucggcucgaguua
NW_002784743.1_11596	3.90E+05	773543	isc-miR-10	uacccuguagauccgaauuugu
NW_002651209.1_28145	3.60E+05	706969	isc-miR-2001	uugugaccguuacaaugggcaug
NW_002835324.1_5031	1.80E+05	356093	isc-miR-12	ugaguauuacaucagguacuggu
NW_002509761.1_45916	1.60E+05	327576	isc-bantam	ugagaucauugugaaagcugauu
NW_002505804.1_46394	1.60E+05	327576	isc-bantam	ugagaucauugugaaagcugauu
NW_002871802.1_369	4.90E+04	96352	isc-miR-100	aacccguagauccgaacuugug
NW_002656291.1_27550	4.10E+04	81041	isc-miR-3931	uacuuugagucgguacgaauccu
NW_002648701.1_28526	3.60E+04	70402	isc-miR-8	uaauacugucagguaaagauguc
NW_002835324.1_5033	3.40E+04	66800	isc-miR-5307	uaaucucauuugguaucucuggg
NW_002778756.1_12266	2.80E+04	54755	isc-miR-79	ucuuugguuaucuagcuguauga
NW_002527183.1_43986	2.20E+04	43978	isc-miR-263a	aauggcacuggaagaauucacgg
NW_002527216.1_43977	2.20E+04	42981	isc-miR-279	ugacuagauccacacucaucca
NW_002741283.1_17066	1.50E+04	29423	isc-miR-276	uaggaacuucauaccaugcucg
NW_002822737.1_6809	1.40E+04	25686	isc-miR-87	gugcccggaacuuugucucagccu
NW_002697059.1_22632	1.30E+04	25752	isc-miR-184	uggacggagaacugauaagggc
NW_002784743.1_11606	9.80E+03	19228	isc-miR-317	ugaacacagcuggugguauaucagu
NW_002838206.1_4665	8.70E+03	16890	isc-miR-305	auuguacuucaucaggugcucugga

NW_002692816.1_23154	6.90E+03	13241	isc-miR-2b	uaucacagccaccuuugaugagcu
NW_002692816.1_23150	5.60E+03	10571	isc-miR-2a	uaucacagccagcuuugaugagc
NW_002692816.1_23156	5.50E+03	7193	isc-miR-71	ucucacuaccuugucuuuguug
NW_002804504.1_9143	4.60E+03	5941	isc-miR-307	ccucacucaguuuggcuguggug
NW_002535453.1_43180	3.00E+03	5915	isc-miR-153	uugcauagucacaaaagugaug
NW_002764570.1_13794	2.80E+03	5474	isc-miR-252b	uuaaguaguagugccgcagguaa
NW_002604667.1_33926	2.80E+03	5463	isc-miR-315	uuuugauuguugcucagaaggcg
NW_002704625.1_21791	1.80E+03	3647	isc-miR-1	uggaauguaaagaaguauggag
NW_002734393.1_17986	1.00E+03	1901	isc-miR-278	ccggaugaauuucucgccuggcc
NW_002848014.1_3640	6.90E+02	1368	isc-miR-5314	uauagaugaugucuucaugaug
NW_002627241.1_30834	6.60E+02	1302	isc-miR-5315	aacacaacauccggacaagcac
NW_002527183.1_43990	3.70E+02	575	isc-miR-96	uuuggcacuagcacauuuuugu
NW_002522955.1_44449	3.10E+02	434	isc-miR-7	uggaagacuagugauuuuguuguu
NW_002784743.1_11634	2.10E+02	330	isc-miR-993	gaagcucguuucuacagguaucu
NW_002805128.1_9069	1.00E+02	90	isc-miR-5308	ucugugcuguggagguaauauau
NW_002589297.1_36170	9.40E+01	76	isc-miR-5305	uaaguuaaucuccaagcccaau
NW_002733905.1_18011	5.3	272	isc-miR-124	uaaggcacgcggugaaugcc
NW_002506863.1_46298	5.3	1270	isc-miR-133	uugguccccuucaaccagcugu
NW_002542293.1_42026	2.1	147	isc-miR-5310	uguagucuggcagaaacguc
NW_002610401.1_33095	2	69	isc-miR-5306	agaguaucacgugacgcuccu
NW_002755260.1_15039	2	28	isc-miR-1993	cauuaugcuaguguucgcgggg
NW_002796881.1_10053	1.9	28	isc-miR-1993	cauuaugcuaguguucgcgggg
NW_002638804.1_29568	1.8	693	isc-miR-5309	caaugcccauggaaaccccgaa
NW_002793850.1_10420	1.7	743	isc-miR-5312	uggcugaacguuguuaugcgu
NW_002838206.1_4667	1.2	17097	isc-miR-275	ucagguaccugaaguagcgcgc
NW_002784743.1_11605	1.1	19228	isc-miR-317	ugaacacagcuggugguauaucagu
NW_002822737.1_6807	-0.8	3011	isc-miR-87	gugagcaaaguuucaggugugu
NW_002796881.1_10054	-2.2	28	isc-miR-1993	cauuaugcuaguguucgcgggg
NW_002741199.1_17073	-5.1	12	isc-miR-137	uuauugcuugagaauacacgu

Table S2. KEGG pathways targeted by differentially expressed miRNAs.

Target proteins	KEGG ID	KEGG pathways
EEC07251.1	KEGG:R02541	RAS-related protein, putative, Sphingolipid metabolism pathway
EEC06395.1	KEGG:R04030	Ubiquinone and other terpenoid-quinone biosynthesis,
		Metabolic pathways
		Biosynthesis of secondary metabolites
		Biosynthesis of cofactors
EEC17038.1	KEGG:R02541	
EEC08267.1	KEGG:R00253	Arginine biosynthesis
		Alanine, aspartate and glutamate metabolism
		Glyoxylate and dicarboxylate metabolism
		Nitrogen metabolism
		Metabolic pathways
		Microbial metabolism in diverse environments
		Biosynthesis of amino acids
EEC06395.1	KEGG:R04030	Fatty-acyl-CoA Synthase (Fatty acid biosynthesis pathway)
EEC17038.1	KEGG:R02541	RAB-33, putative [Ixodes scapularis], exosome biogenesis and secretion between arthropods and mammals.
EEC08267.1	KEGG:R00253	
EEC10220.1	KEGG:R04188	4-aminobutyrate aminotransferase, Valine, leucine and isoleucine degradation
EEC02614.1	KEGG:R01178	medium-chain acyl-CoA dehydrogenase, putative, Lipid transport and metabolism
EEC07988.1	KEGG:R05982<br>KEGG:R06722	alpha-mannosidase putative, Protein processing in endoplasmic reticulum, Various types of N-glycan biosynthesis

EEC02307.1	KEGG:R01178	medium-chain acyl-CoA dehydrogenase, putative,
EEC07756.1	KEGG:R03876	ubiquitin protein ligase, putative,
EEC20239.1	KEGG:R03532	thioredoxin-dependent peroxide reductase, which confer a protective role in cells through its peroxidase activity by reducing hydrogen peroxide
EEC04717.1	KEGG:R03876	RNA polymerase II transcription elongation factor, putative,
EEC17038.1	KEGG:R02541	RAB-33, putative [Ixodes scapularis], exosome biogenesis and secretion between arthropods and mammals.
EEC09178.1	KEGG:R07364	acireductone dioxygenase, putative, Cysteine and methionine metabolism
EEC17038.1	KEGG:R02541	RAB-33, putative [Ixodes scapularis], exosome biogenesis and secretion between arthropods and mammals.
EEC08267.1	KEGG:R00253	glutamine synthetase, putative,
EEC09478.1	KEGG:R02324	nicotinamide riboside kinase, putative, Nicotinate and nicotinamide metabolism
EEC06118.1	KEGG:R02530	4-hydroxyphenylpyruvate dioxygenase, putative, Pyruvate metabolism
EEC07756.1	KEGG:R03876	ubiquitin protein ligase, putative,
EEC07147.1	KEGG:R05330	short-chain dehydrogenase, putative, Primary bile acid biosynthesis, Biosynthesis of unsaturated fatty acids
EEC11438.1	KEGG:R02541	RAS-related protein, putative, exosome biogenesis and secretion between arthropods and mammals
EEC08160.1	KEGG:R02541	GTP-binding protein Rhes, putative, exosome biogenesis and secretion between arthropods and mammals.
EEC10220.1	KEGG:R04188	4-aminobutyrate aminotransferase, Valine, leucine and isoleucine degradation
EEC07981.1	KEGG:R01049	ribose-phosphate pyrophosphokinase 1 putative,
		rn00030 Pentose phosphate pathway<br>rn00230 Purine metabolism<br>rn01100 Metabolic pathways<br>rn01110 Biosynthesis of secondary metabolites<br>rn01120 Microbial metabolism in diverse environments<br>rn01200 Carbon metabolism<br>rn01230 Biosynthesis of amino acids
EEC08267.1	KEGG:R00253	glutamine synthetase putative, Arginine biosynthesis, Alanine, aspartate and glutamate metabolism, Glyoxylate and dicarboxylate, nitrogen metabolism, Microbial metabolism in diverse environments, Biosynthesis of amino acids
EEC05869.1	KEGG:R02268	cytochrome P450 putative, Arachidonic acid metabolism
EEC04717.1	KEGG:R03876	RNA polymerase II transcription elongation factor, putative
EEC08267.1	KEGG:R00253	glutamine synthetase putative, Arginine biosynthesis, Alanine, aspartate and glutamate metabolism, Glyoxylate and dicarboxylate, nitrogen metabolism, Microbial metabolism in diverse environments, Biosynthesis of amino acids
EEC07251.1	KEGG:R02541	RAS-related protein putative, exosome biogenesis and secretion between arthropods and mammals
EEC17749.1	KEGG:R02541	RAB-9 and, exosome biogenesis and secretion between arthropods and mammals
EEC09478.1	KEGG:R02324	nicotinamide riboside kinase (putative), Nicotinate and nicotinamide metabolism
EEC05808.1	KEGG:R03876	
EEC02290.1	KEGG:R00004	secreted inorganic pyrophosphatase, putative,
EEC15523.1	KEGG:R02265	Microsomal prostaglandin E synthase 2, Arachidonic acid metabolism
EEC08267.1	KEGG:R00253	glutamine synthetase putative, Arginine biosynthesis, Alanine, aspartate and glutamate metabolism, Glyoxylate and dicarboxylate, nitrogen metabolism, Microbial metabolism in diverse environments, Biosynthesis of amino acids
EEC10220.1	KEGG:R04188	(S)-3-amino-2-methylpropionate transaminase, Valine, leucine and isoleucine degradation
EEC01042.1	KEGG:R00310	Ferrochelatase, Porphyrin and chlorophyll metabolism, Biosynthesis of secondary metabolites, Heme biosynthesis

Table S3. Gene-specific PCR and qRT-PCR primers used in this study.

Gene	GenBank ID	Forward Primer (5'-3')	Reverse Primer (5'-3')	Size (bp)
RPS4 (qPCR)	DQ066214.1	GGTGAAGAAGATTGTCAAGCAGAG	TGAAGCCAGCAGGGTAGTTTG	80
flaB (qPCR)	Stone et al., 2015	GGG TCT CAA GCG TCT TGG	GAA CCG GTG CAG CCT GAG	139
